# RETRACTION: Role of Matrix Metalloproteinase‐9 Gene Polymorphisms in Glaucoma: A Hospital‐Based Study in Chinese Patients

**DOI:** 10.1002/jcla.70094

**Published:** 2025-08-28

## Abstract

RETRACTION: ZhaoF. FanZ. HuangX. Role of Matrix Metalloproteinase‐9 Gene Polymorphisms in Glaucoma: A Hospital‐Based Study in Chinese Patients Journal of Clinical Laboratory Analysis 34 no. 3 (2020): e23105, 10.1002/jcla.23105 31713905 PMC7083395

The above article, published online on 12 November 2019 in Wiley Online Library (wileyonlinelibrary.com), has been retracted by agreement between the journal Editor‐in‐Chief, Rong Fu; and Wiley Periodicals LLC. The retraction has been agreed due to scientific inconsistencies and major flaws in the statistical evaluation.

The journal received a request to retract this manuscript initially. During investigation, the authors stated that original data is no longer available and did not provide documentation for the ethical approval process of this study. Nanchong Central Hospital could not be reached for comment. Subsequently, the authors stopped responding.

The conclusions of this manuscript are considered unreliable.

RETRACTION: Zhao F. Fan Z. Huang X. Role of Matrix Metalloproteinase‐9 Gene Polymorphisms in Glaucoma: A Hospital‐Based Study in Chinese Patients Journal of Clinical Laboratory Analysis 34 no. 3 (2020): e23105, 10.1002/jcla.23105 31713905 PMC7083395

The above article, published online on 12 November 2019 in Wiley Online Library (wileyonlinelibrary.com), has been retracted by agreement between the journal Editor‐in‐Chief, Rong Fu; and Wiley Periodicals LLC. The retraction has been agreed due to scientific inconsistencies and major flaws in the statistical evaluation.

The journal received a request to retract this manuscript initially. During investigation, the authors stated that original data is no longer available and did not provide documentation for the ethical approval process of this study. Nanchong Central Hospital could not be reached for comment. Subsequently, the authors stopped responding.

The conclusions of this manuscript are considered unreliable.

